# Phosphorylated TDP-43 aggregates in skeletal and cardiac muscle are a marker of myogenic degeneration in amyotrophic lateral sclerosis and various conditions

**DOI:** 10.1186/s40478-019-0824-1

**Published:** 2019-10-28

**Authors:** Fumiaki Mori, Mari Tada, Tomoya Kon, Yasuo Miki, Kunikazu Tanji, Hidekachi Kurotaki, Masahiko Tomiyama, Tomohiko Ishihara, Osamu Onodera, Akiyoshi Kakita, Koichi Wakabayashi

**Affiliations:** 1 Department of Neuropathology, Institute of Brain Science, Hirosaki University Graduate School of Medicine, 5 Zaifu-cho, Hirosaki, 036-8562 Japan; 2 Department of Pathology, Brain Research Institute, Niigata University, Niigata, Japan; 3 Department of Neurology, Hirosaki University Graduate School of Medicine, Hirosaki, Japan; 4 Department of Pathology, Aomori Prefectural Central Hospital, Aomori, Japan; 5 Department of Molecular Neuroscience, Brain Research Institute, Niigata University, Niigata, Japan; 6 Department of Neurology, Brain Research Institute, Niigata University, Niigata, Japan

**Keywords:** Amyotrophic lateral sclerosis, Myocardium, Myogenic degeneration, Skeletal muscle, TDP-43

## Abstract

**Background:**

Amyotrophic lateral sclerosis (ALS) is characterized pathologically by the occurrence of phosphorylated TDP-43 (pTDP-43)-immunoreactive neuronal and glial inclusions in the central nervous system. Recent studies have shown that pTDP-43 aggregates also occur in the skeletal muscles in a certain proportion of ALS patients.

**Aim:**

The aim of this study was to clarify the distribution and incidence of pTDP-43 aggregates in the skeletal and cardiac muscles of patients with ALS, and also those of patients with neuromuscular diseases (NMDs) and non-NMDs.

**Material and methods:**

Five regions of muscle (tongue, cervical muscle, diaphragm, iliopsoas muscle and heart) were examined histologically and immunohistochemically in patients with ALS (*n* = 30), NMDs (*n* = 13) and non-NMDs (*n* = 7).

**Results:**

Two types of pTDP-43 aggregates were distinguishable morphologically: dense filamentous and short linear inclusions. These inclusions were found in at least one of the five muscle regions in all 30 cases of ALS; skeletal muscles in 28 cases and myocardium in 12. pTDP-43 aggregates were also found in 9 of 13 patients with NMDs, including myositis, muscular dystrophy and mitochondrial myopathy, as well as in 3 of 7 patients with non-NMDs. In ALS, pTDP-43 aggregates were most frequent in the diaphragm (19 cases). The mean density of pTDP-43 aggregates in ALS was significantly higher than that in NMDs and non-NMDs. In contiguous sections stained with hematoxylin and eosin and anti-pTDP-43, muscle fibers with dense filamentous inclusions demonstrated single-fiber atrophy with vacuolar degeneration.

**Conclusion:**

The present findings indicate that pTDP-43 aggregates in skeletal and cardiac muscle are a myogenic pathological marker in multiple diseases including ALS.

## Introduction

Amyotrophic lateral sclerosis (ALS) is characterized pathologically by loss of upper and lower motor neurons with consistent occurrence of phosphorylated TDP-43 (pTDP-43)-immunoreactive neuronal and glial inclusions in the central nervous system [2, 15]. Although neurogenic atrophy is found in the skeletal muscles, no pTDP-43 aggregates have been reported in the quadriceps [20], deltoid [1] or gastrocnemius muscles [23] in patients with ALS.

However, TDP-43 aggregates have been described in the skeletal muscles from patients with various diseases, including sporadic inclusion body myositis (IBM) [7, 26], ALS with TARDBP mutation [8], IBM with Paget’s disease of the bone and fronto-temporal dementia [3, 25], polymyositis with mitochondrial pathology [22], non-IBM myopathies with rimmed vacuoles (limb girdle muscular dystrophy and myotonic dystrophy type 2) [9], distal hereditary motor neuropathy and myofibrillary myopathy with HSPB8 mutation [4], limb girdle muscular dystrophy with DNAJB6 mutation [19], myofibrillar myopathies (myotilinopathy and desminopathies) [17], oculopharyngeal muscular dystrophy and distal myopathy with rimmed vacuoles and GNE mutation [10] and hereditary motor and sensory neuropathy [27]. Thus, the number of neuromuscular diseases (NMDs) with TDP-43 inclusions in the skeletal muscles has been increased. Moreover, Vogler et al. reported that TDP-43 and RNA form amyloid-like myo-granules in regenerating muscle fibers, suggesting that myo-granules could be the source of cytoplasmic TDP-43 aggregates [24].

Recently, Cykowski et al. demonstrated pTDP-43 aggregates in the skeletal muscles of 19 among 57 patients (33.3%) with ALS, including sporadic and familial cases, being more common in the axial muscles (paraspinous muscles and diaphragm) than in the appendicular muscles [6]. Among 25 non-ALS muscle samples, pTDP-43 aggregates were seen only in four patients with IBM.

The growing list of reports on TDP-43 aggregates in the skeletal muscles from patients with ALS and various NMDs prompted us to investigate the significance of pTDP-43 aggregates as a pathological marker in muscle tissue. Using immunohistochemistry, we examined the skeletal and cardiac muscles of patients with sporadic ALS, and also patients with NMDs and non-NMDs. Here we report that pTDP-43 aggregates are present in both skeletal and cardiac muscles in various conditions, including ALS as well as NMDs and non-NMDs, and that these aggregates are associated with myogenic muscle degeneration.

## Materials and methods

### Subjects

Our investigations were performed using a two-step approach. First, 27 autopsy cases (Group A) were examined to investigate whether pTDP-43 aggregates are present in cardiac muscle; these included cases of sporadic ALS (patient age 52–80 years, average = 67.9 years; *n* = 16), NMDs (patient age 49–85 years, average = 65.4 years; *n* = 5), and non-NMDs (patient age 35–84 years, average = 63.7 years; *n* = 6) (Table 1). Tissue samples were obtained from the Department of Neuropathology, Hirosaki University Graduate School of Medicine. For routine histological examination, 4-μm-thick, formalin-fixed, paraffin-embedded sections from the brain and spinal cord were stained with hematoxylin and eosin (HE) or by the Klüver-Barrera method. The pathological diagnosis of ALS had been confirmed by neuropathological examinations using immunohistochemistry for pTDP-43. We examined at least one of five muscle regions (tongue, cervical muscle, diaphragm, iliopsoas muscle and myocardium) in each case. These examinations revealed the presence of pTDP-43 aggregates in various muscle regions in ALS, NMDs and non-NMDs, as described in the Results section.

**Table 1:**
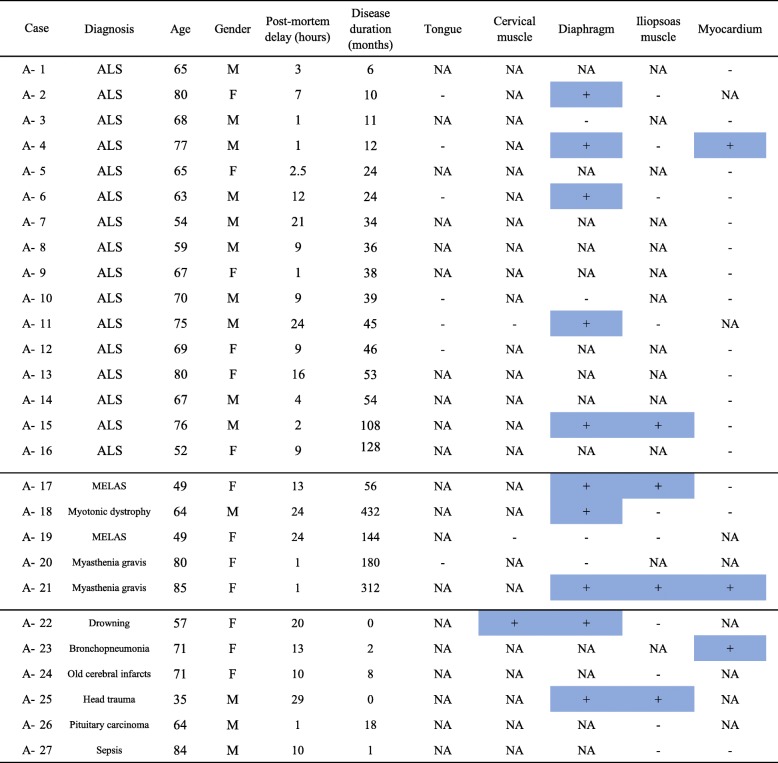
Distribution of phosphorylated TDP-43 aggregates in Group A

Phosphorylated TDP-43 aggregates: +, present; −, absent; *NA* Not available

*ALS* Amyotrophic lateral sclerosis, *MELAS* Mitochondrial encephalomyopathy, lactic acidosis and stroke-like episodes

Second, histological and immunohistochemical investigations were conducted to clarify the muscle pathology and the distribution, incidence and density of pTDP-43 aggregates in the skeletal and cardiac muscles. Fifty autopsy cases were investigated in this second study (Group B); these included cases of sporadic ALS (patient age 57–86 years, average = 70.9 years; *n* = 30), NMDs (patient age 25–83 years, average = 57.2 years; *n* = 13), and non-NMDs (patient age 47–87 years, average = 68.9 years; *n* = 7) (Table 2). Among the cases of sporadic ALS, 15 were Nishihira type 1 and the remainder had Nishihira type 2 pathology [16]. Tissue samples were obtained from the Department of Pathology, Brain Research Institute, Niigata University. We examined all of the five muscle regions (tongue, cervical muscle, diaphragm, iliopsoas muscle and myocardium) in each case.

**Table 2:**
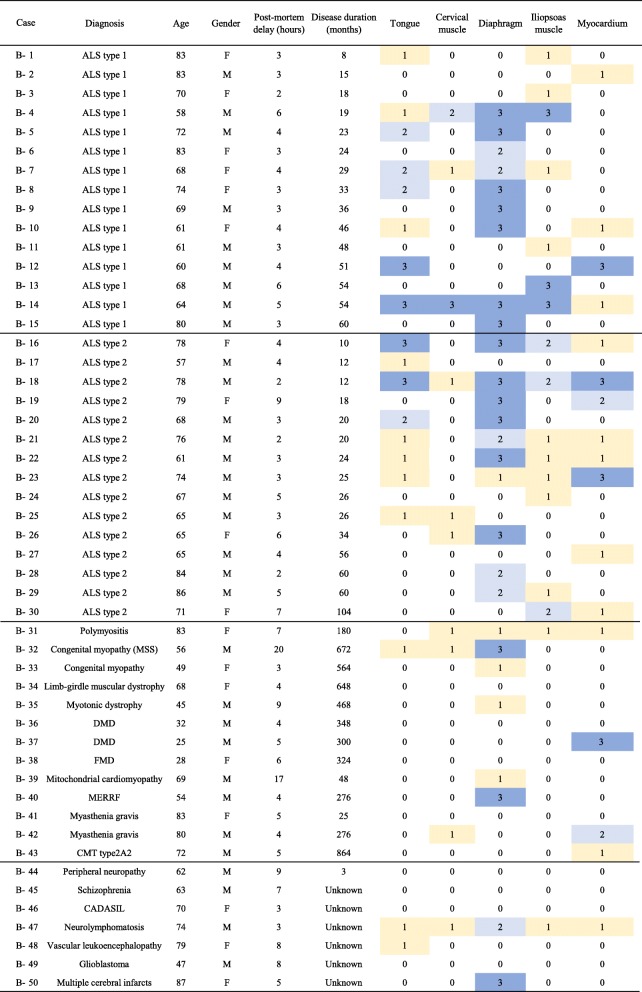
Distribution and density of phosphorylated TDP-43 aggregates in Group B

Density of phosphorylated TDP-43 aggregates (number / cm^2^): “0”, 0; “1”, < 1 (yellow); “2”, 1–2 (light blue); “3”, > 2 (blue)

*ALS* Amyotrophic lateral sclerosis, *MSS* Marinesco-Sjögren syndrome, *DMD* Duchenne muscular dystrophy, *FMD* Fukuyama-type musular dystrophy, *MERRF* Myoclonus epilepsy associated with ragged-red fibers, *CADASIL* Cerebral autosomal dominant arteriopathy with subcortical infarcts and leukoencephalopathy, *CMT* Charcot-Marie-Tooth disease

### Immunohistochemistry

Four-micrometer-thick, formalin-fixed, paraffin-embedded sections of skeletal muscles and heart were subjected to immunohistochemical processing using the avidin-biotin-peroxidase complex method with diaminobenzidine as the chromogen. The primary antibodies used were rabbit polyclonal antibodies against pTDP-43 (pSer409/410; Cosmo Bio Co., Ltd., Tokyo, Japan; 1:5000), native TDP-43 (nTDP-43; 10,782–1-AP; ProteinTec Group, Inc., Chicago, IL, USA; 1:5000) and p62 (BD Biosciences, Franklin Lakes, NJ, USA; 1:100). The sections were pretreated in an autoclave for 15 min in 10 mM citrate buffer (pH 6.0).

To evaluate whether pTDP-43 aggregates are proteinase K (PK)-resistant, PK (Gibco BRL, Gaithersburg, MD, USA; 50 mg/mL) in PK buffer (10 mM Tris-HCl, pH 7.8, 100 mM NaCl, 0.1% Nonidet-P40) at 37 °C for 10 min was applied to selected sections.

### Semi-quantitative analysis of pTDP-43 pathology in muscles

We developed a semi-quantitative scale to score the density of pTDP-43 aggregates in skeletal and cardiac muscles. The total number of pTDP-43 aggregates was quantified in each section. The entire regions were surveyed at × 200 magnification using an eyepiece graticule and parallel sweeps of the microscope stage. We measured the whole area of each section using Image J software provided by the National Institutes of Health and calculated the density of pTDP-43 aggregates in each section (“0”, not detectable; “1”, detectable in < 1 pTDP-43 aggregate per 1 cm^2^ of section; “2”, detectable in 1–2 pTDP-43 aggregates per 1 cm^2^ of section; and “3”, detectable in > 2 pTDP-43 aggregates per 1 cm^2^ of section).

### Muscle pathology

Contiguous sections were stained with HE and anti-pTDP-43 in the second study. Presence or absence of muscle pathology (neurogenic atrophy, myogenic atrophy or single-fiber atrophy with vacuolar degeneration) was investigated in each section.

### Statistical analysis

To determine whether pTDP-43 aggregates are more common to be found in ALS than in non-ALS groups (NMDs and non-NMDs), Kruskal-Wallis and Steel-Dwass tests were applied to differences in the density of pTDP-43 aggregates between the groups. To determine which region is more vulnerable to pTDP-43 pathology, Quade and Steel-Dwass tests were applied to differences in the density of pTDP-43 aggregates between the five muscle regions. Calculations were performed using Statcel software (OMS Publishing, Tokorozawa, Japan).

### Analysis of the TARDBP and C9ORF72 genes

As for ALS cases in Group B, the presence or absence of TARDBP and C9ORF72 gene mutations was analyzed in 29 cases for which frozen tissue samples were available (other than B-30) as described previously [21]. ALS cases in Group A and non-ALS cases in both groups were not genetically assessed for ALS related genes.

## Results

### Morphology of pTDP-43 aggregates in muscles

**Fig. 1:**
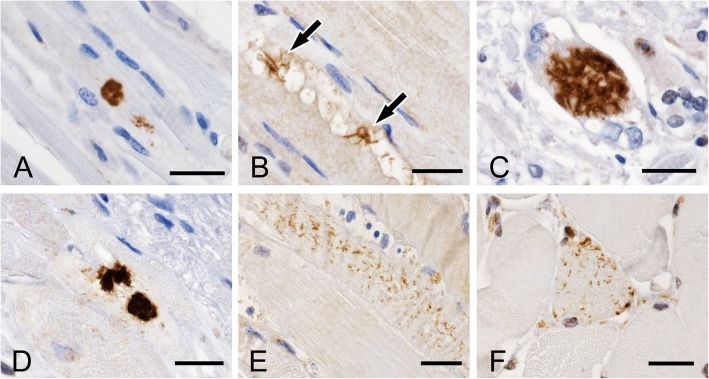
Representative findings of pTDP-43 immunohistochemistry in skeletal and cardiac muscles. **a**-**d** Dense filamentous (round or stellate) inclusions in the diaphragm (**a** and **b**), iliopsoas muscle (**c**) and myocardium (**d**) in patients with ALS (**a** case A-6; **b** case B-5; **c** case A-15; **d** case A-4). **e** and **f** Short linear inclusions in the diaphragm of a patient with ALS (**e** case B-16) and in the cervical muscle of a patient with non-neuromuscular disease (**f** case B-47). Bars = 20 μm

Immunostaining with anti-pTDP-43 antibody revealed pTDP-43 aggregates in fibers of skeletal muscles (tongue, cervical muscle, diaphragm and iliopsoas muscle) and cardiac muscle. Two types of pTDP-43 aggregates were distinguishable morphologically: dense filamentous (Fig. 1a-d) and short linear (Fig. 1e, f) inclusions.

### Distribution and incidence of pTDP-43 aggregates in muscles

pTDP-43 aggregates were found in at least one region of skeletal or cardiac muscle in 5 of 16 cases of ALS (31.3%), 3 of 5 cases of NMDs (60%), and 3 of 6 cases of non- NMDs (50%) in Group A (Table 1). Histological and immunohistochemical investigations were then conducted to clarify the distribution and incidence of pTDP-43 aggregates in skeletal and cardiac muscle.

In each of the 50 cases in Group B, 5 regions (tongue, cervical muscle, diaphragm, iliopsoas muscle and myocardium) were examined (Table 2). pTDP-43 aggregates were found in at least one of these 5 regions in all 30 cases of ALS (100%); pTDP-43 aggregates were present in the skeletal muscles of 28 cases and in the myocardium in 12 (Table 2). pTDP-43 aggregates were also found in 9 of the 13 patients with NMDs (69.2%) and in 3 of the 7 patients with non-NMDs (42.9%) (Table 2). In the ALS group, pTDP-43 aggregates were present most frequently in the diaphragm (19 cases). However, they were also found in the tongue (16 cases), iliopsoas muscle (15 cases), myocardium (12 cases) and cervical muscle (6 cases). In the patients with NMDs, pTDP-43 aggregates were found in the diaphragm (6 cases), myocardium (4 cases), cervical muscle (3 cases), tongue (1 case) and iliopsoas muscle (1 case). These positive cases included polymyositis, Marinesco-Sjögren syndrome, congenital myopathy, myotonic dystrophy, Duchenne muscular dystrophy, mitochondrial cardiomyopathy, myoclonus epilepsy associated with ragged-red fibers, myasthenia gravis and Charcot-Marie-Tooth disease type2A2. In the patients with non-NMDs, pTDP-43 aggregates were found in the tongue (2 cases), diaphragm (2 cases), cervical muscle (1 case), iliopsoas muscle (1 case) and myocardium (1 case). These positive cases included neurolymphomatosis, vascular leukoencephalopathy and multiple cerebral infarction. pTDP-43 aggregates were not noted in the peripheral nerve elements in any of the patients with ALS, NMDs or non-NMDs.

### Density of pTDP-43 aggregates in muscles

**Fig. 2:**
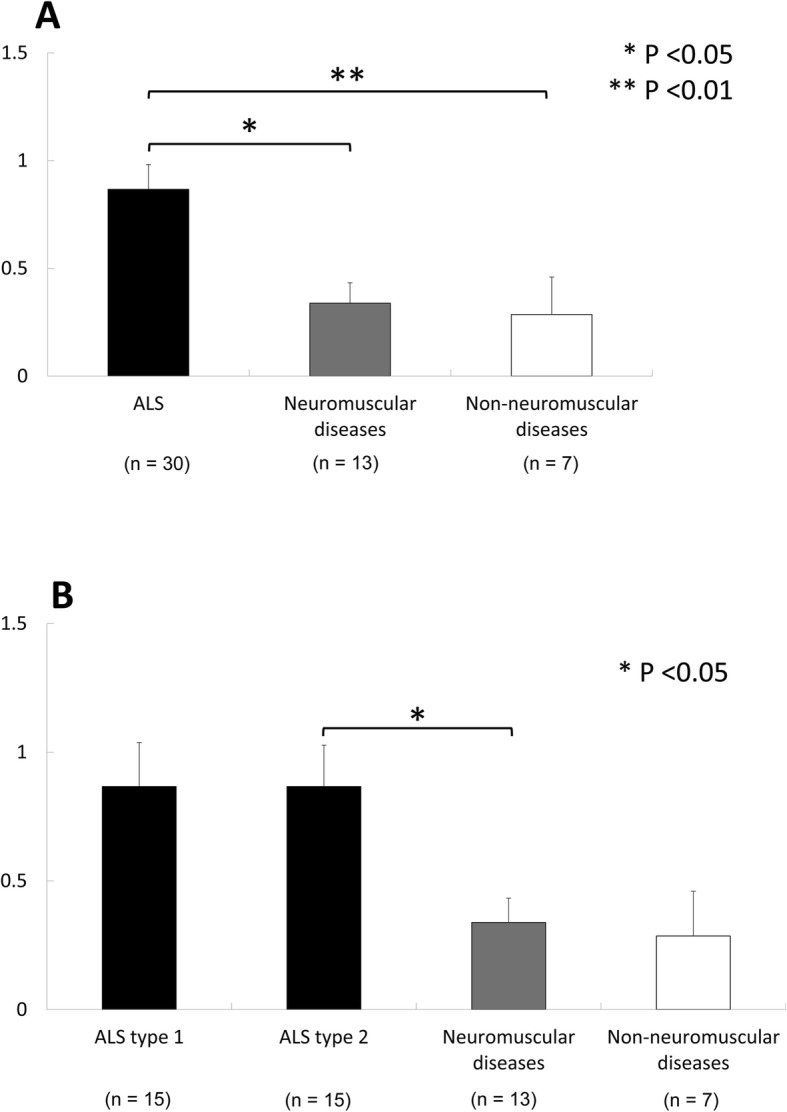
Comparison of the mean density of pTDP-43 aggregates among patients with ALS, neuromuscular diseases and non-neuromuscular diseases (**a**). Comparison among patients with ALS types 1 and 2, neuromuscular diseases and non-neuromuscular diseases (**b**)

Since the incidence of pTDP-43 aggregates in ALS was higher than that in NMDs and non-NMDs, we statistically compared the density of pTDP-43 aggregates between these three groups. The mean density of pTDP-43 aggregates in ALS was significantly higher than that in NMDs and non-NMDs (Fig. 2a). The mean density of pTDP-43 aggregates in ALS types 1 and 2 was also significantly higher than that in non-NMDs (Fig. 2b).

**Fig. 3:**
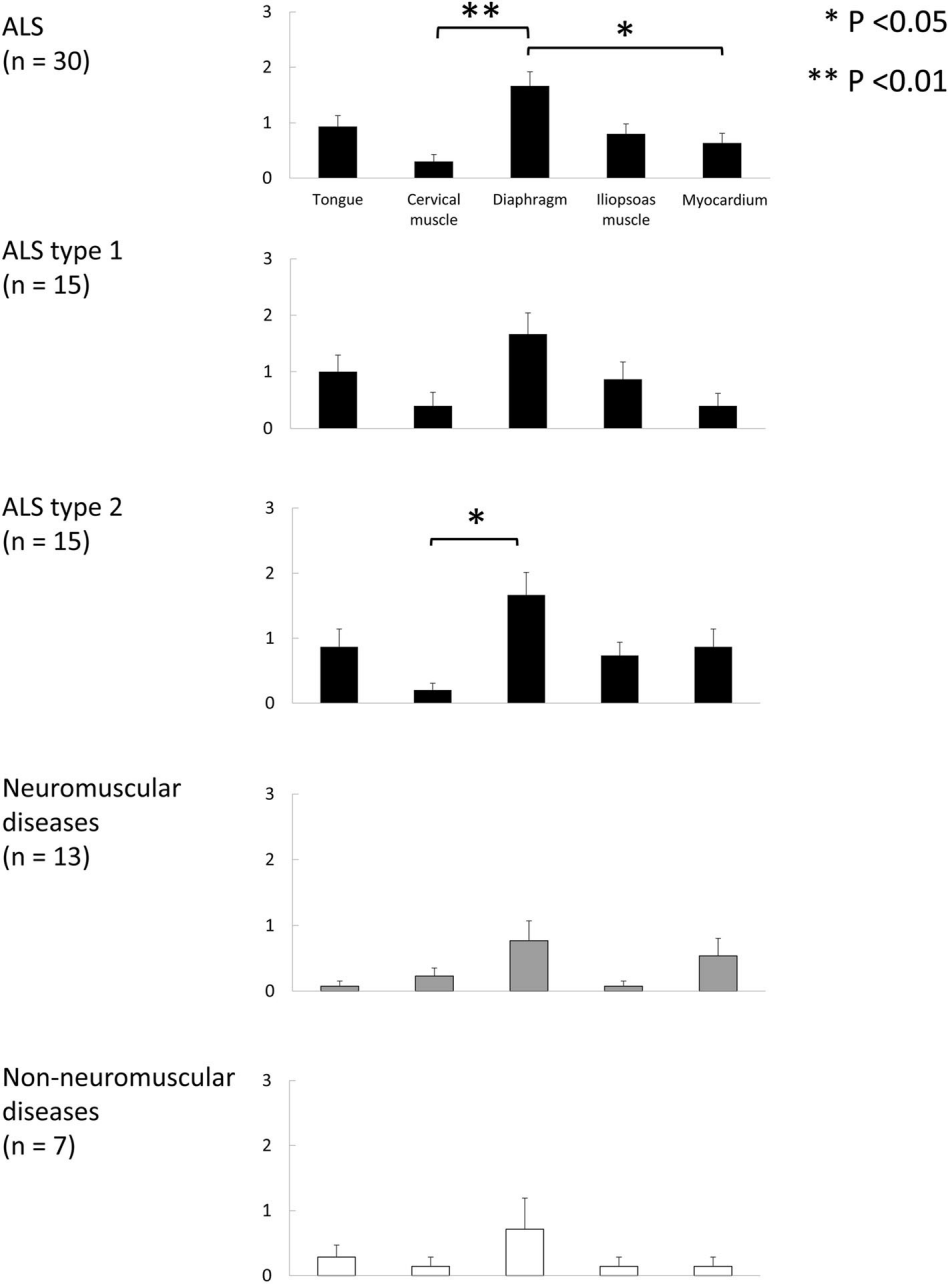
Comparison of the mean density of pTDP-43 aggregates among the tongue, cervical muscle, diaphragm, iliopsoas muscle and myocardium in each group of patients with ALS, ALS types 1 and 2, neuromuscular diseases and non-neuromuscular diseases

We further compared the density of pTDP-43 aggregates in five types of muscle. The mean density of pTDP-43 aggregates in the diaphragm was significantly higher than that in the cervical muscle and myocardium in ALS (Fig. 3). The mean density of pTDP-43 aggregates in the diaphragm in ALS type 2 was significantly higher than that in the cervical muscle (Fig. 3). No significant regional differences were evident in NMDs and non-NMDs.

### Immunohistochemical features of pTDP-43 aggregates in muscles

**Fig. 4:**
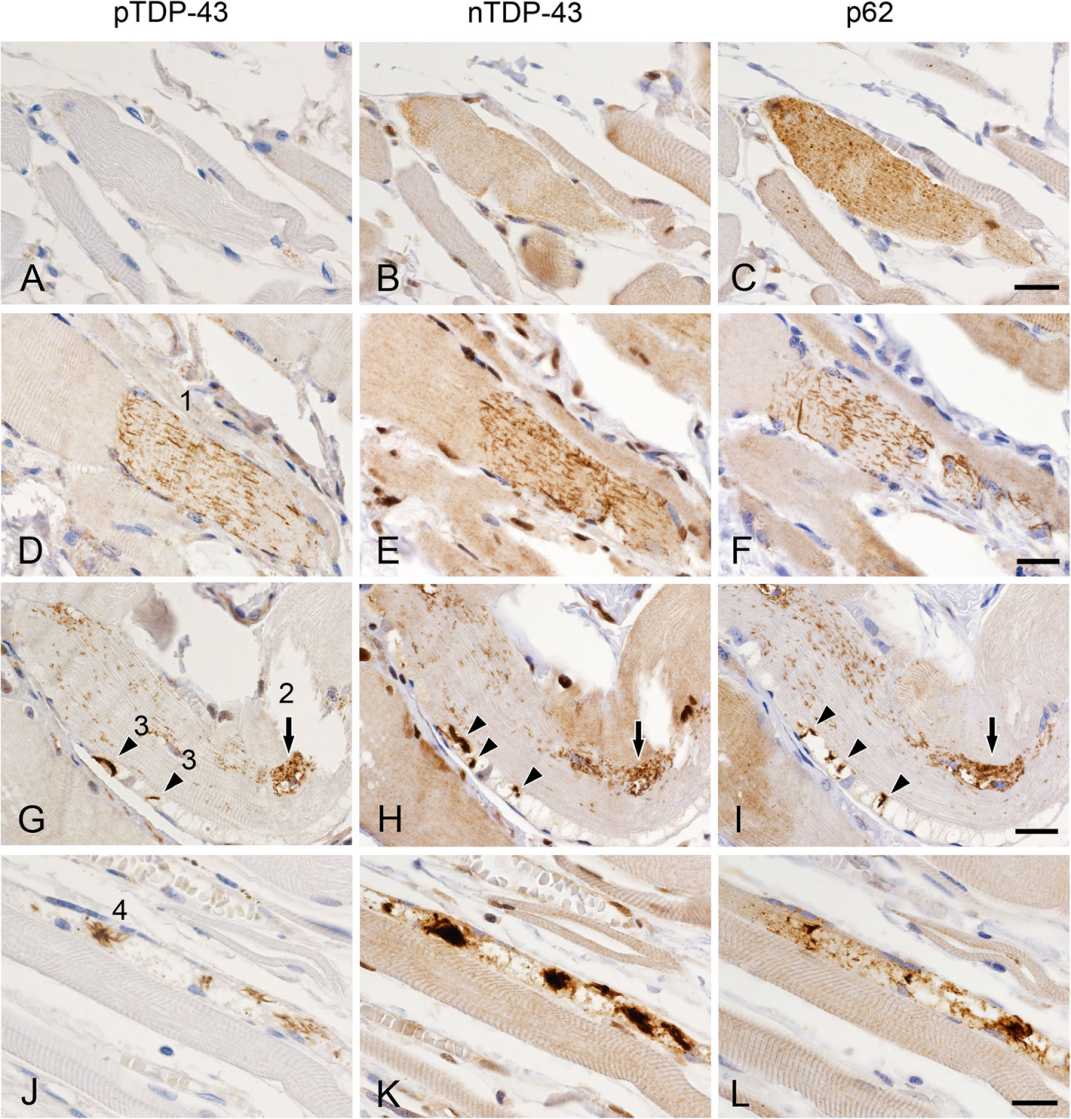
Immunohistochemistry for pTDP-43 (**a**, **d**, **g** and **j**), nTDP-43 (**b**, **e**, **h** and **k**) and p62 (**c**, **f**, **i** and **l**) in serial sections of myofibers in the diaphragm from patients with ALS. **a**-**c** Normal-looking myofibers containing fine granular structures immunopositive for nTDP-43 (**b**) and p62 (**c**), but not for pTDP-43 (**a**) (case A-6). **d**-**f** Short linear inclusions are immunopositive for pTDP-43 (1 in **d**), nTDP-43 (**e**) and p62 (**f**) (case B-16). **g**-**i** Accumulated short linear inclusions (arrows; 2 in **g**) and small dense filamentous inclusions (arrowheads, 3 in **g**) are immunopositive for pTDP-43 (**g**), nTDP-43 (**h**) and p62 (**i**) (case B-16). **j**-**l** Larger dense filamentous inclusions (4 in **j**) are immunopositive for pTDP-43 (**j**), nTDP-43 (**k**) and p62 (**l**) (case A-6). Bars = 20 μm

Contiguous sections immunolabeled with anti-pTDP-43, anti-nTDP-43 and anti-p62 revealed that pTDP-43-negative normal-looking myofibers were immunopositive for nTDP-43 and p62 (Fig. 4a-c) and that pTDP-43-positive dense filamentous and short linear inclusions were also immunopositive for nTDP-43 and p62 (Fig. 4d-l).

**Fig. 5:**
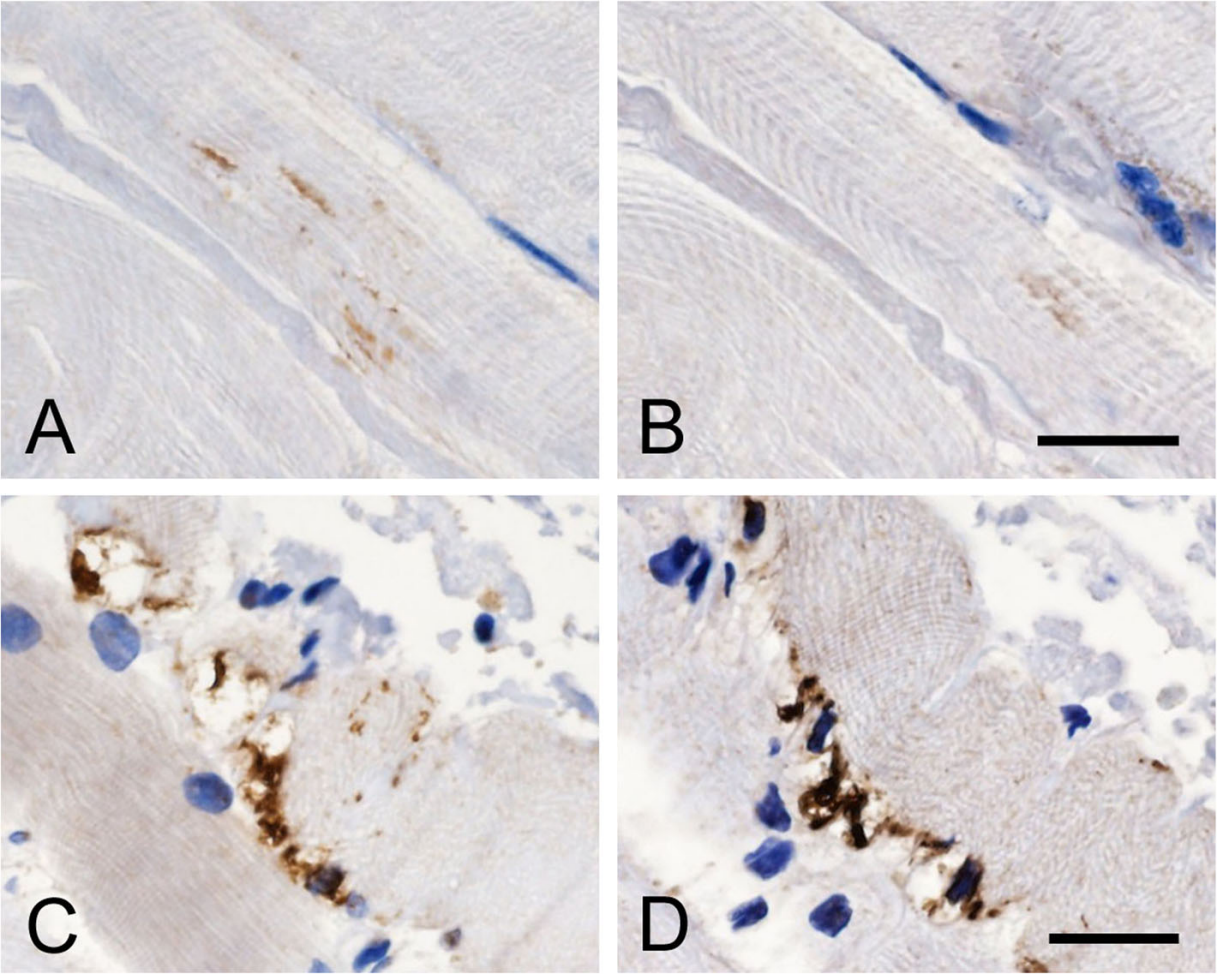
Contiguous sections of the tongue immunostained with anti-pTDP-43 without proteinase K treatment (**a** and **c**) and with proteinase K treatment (**b** and **d**) in a patient with ALS (case B-22). Proteinase K treatment for 10 min decreases the number of short linear inclusions (**a** and **b**), whereas dense filamentous inclusions are proteinase K-resistant (**c** and **d**). Bars = 20 μm

PK treatment for 10 min decreased the number of short linear inclusions (Fig. 5a, b), suggesting that these inclusions are aggregated weakly. On the other hand, dense filamentous inclusions were PK-resistant (Fig. 5c, d), suggesting that these inclusions are aggregated strongly. PK-resistant inclusions were seen in both skeletal muscle and myocardium in patients with ALS, and in those with NMDs and non-NMDs.

### Muscle pathology in cases with and without pTDP-43 aggregates

In the ALS group, various degrees of neurogenic atrophy (grouped atrophy and small angular fibers) were evident in the skeletal muscles in all cases. No myogenic pattern was noted in skeletal muscles, and no obvious alterations were seen in the myocardium.

In the patients with NMDs, myogenic pathology (fiber size variation, myofiber necrosis, internal nuclei, chained nuclei, pyknotic nuclear clumping, fiber splitting and fibrosis) was evident in the skeletal muscles. Moderate to marked fibrosis was seen in the myocardium in 3 cases (cases B-36, − 37 and − 39) and mild fatty degeneration was evident in a case (case B-38).

Among the patients with non-NMDs, mild neurogenic atrophy was seen in the skeletal muscles in a case (case B-44). No obvious changes were evident in the skeletal or cardiac muscles in positive cases (cases B-47, − 48 and − 50).

**Fig. 6:**
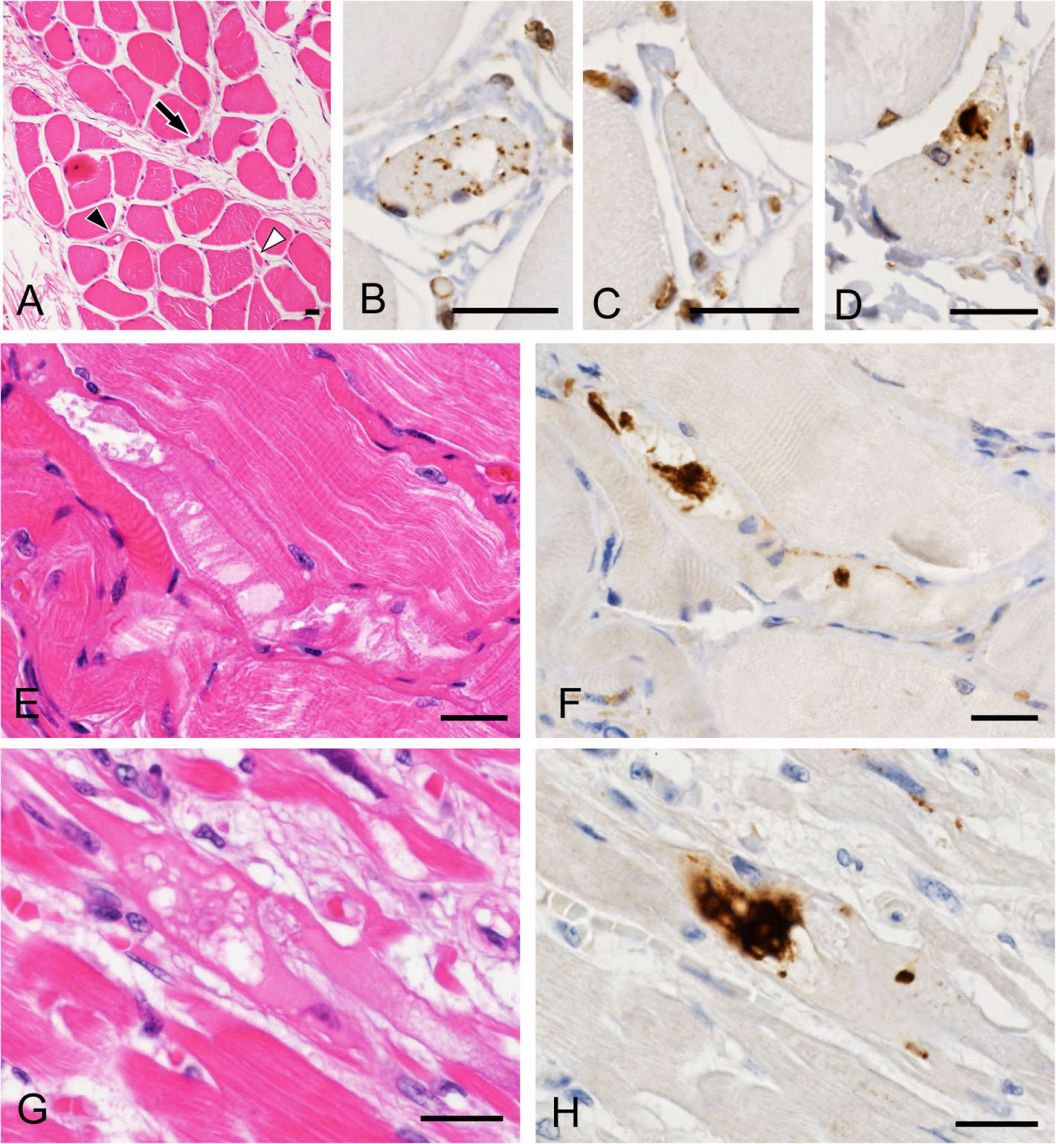
Contiguous sections of the cervical muscle (**a**-**d**), diaphragm (**e** and **f**) and myocardium (**g** and **h**) stained with HE (**a**, **e** and **g**) and anti-pTDP-43 (**b**-**d**, **f** and **h**). **a**-**d** Muscle fibers showing single-fiber atrophy (**a**) contain pTDP-43-positive short linear inclusions (**b** and **c**: black and white arrowheads in (**a**)) and dense filamentous inclusions (**d**: arrow in (**a**)) in a patient with non-neuromuscular disease (case B-47). Note that atrophic fibers contain small vacuoles (**b**). **e** and **f** A muscle fiber showing marked vacuolar degeneration (**e**) contains dense filamentous inclusions immunopositive for pTDP-43 (**f**) in a patient with ALS (case B-16). **g** and **h** A cardiac muscle fiber showing marked vacuolar degeneration (**g**) contains dense filamentous inclusions immunopositive for pTDP-43 (**h**) in a patient with Duchenne muscular dystrophy (case B-37). Bars = 20 μm

In contiguous sections of the skeletal muscles and myocardium stained with HE and anti-pTDP-43, muscle fibers showing single-fiber atrophy contained pTDP-43-positive short linear inclusions and/or dense filamentous inclusions (Fig. 6a-d). Dense filamentous inclusions were also found in muscle fibers showing moderate to marked vacuolar degeneration (Fig. 6e-h).

### Genetic feature

TDP-43 (TARDBP) gene mutation or C9ORF72 repeat expansion was not observed in 29 cases of ALS in Group B.

## Discussion

Recently, Cykowski et al. reported the presence of pTDP-43 aggregates in axial (paraspinous and diaphragm) muscles and appendicular (deltoid and quadriceps) muscles in 19 of 57 patients (33.3%) with sporadic and familial ALS, including C9ORF72 expansion-associated ALS [6]. In the present study, pTDP-43 aggregates were found in at least one of four types of skeletal muscle in 28 of 30 cases of sporadic ALS in Group B (93.3%). The prevalence of pTDP-43 aggregate in skeletal muscles in our ALS patients in Group B (93.3%) was higher than that in Cykowski’s study (33.3%). This difference raises several possibilities, i.e. differences in the number of examined muscles, scoring method, technique and tissue handling etc. In Cykowski’s study, they examined one to four skeletal muscles in each case [6]. In our Group A, we similarly examined one to four skeletal muscles and the prevalence of pTDP-43 aggregates was 62.5% (5 of 8 cases). In our Group B, however, we examined all of four regions (tongue, cervical muscle, diaphragm and iliopsoas muscle) in each case. The difference in the number of examined muscles per patient may explain the difference in the prevalence of pTDP-43 aggregates. Among four regions, pTDP-43 aggregates were most frequent in the diaphragm (19 cases), where the mean density of pTDP-43 aggregates was significantly higher than that in cervical muscle. These findings suggest that skeletal muscles, especially the diaphragm, are especially prone to pTDP-43 pathology in ALS.

Cykowski et al. reported that similar aggregates were also present in patients with IBM, but not in non-ALS patients with neurogenic muscular atrophy [6]. However, TDP-43 aggregates have been described in the skeletal muscles from patients with various NMDs [3, 4, 7–10, 17, 19, 22, 25–27]. In the present study, we examined skeletal muscles from 13 non-ALS patients with NMDs and found pTDP-43 aggregates in 7 of them; the underlying conditions included myositis, myopathy, muscular dystrophy, mitochondrial myopathy, and myasthenia gravis. pTDP-43 aggregates were also found in skeletal muscles in 3 of 7 patients with non-NMDs (malignant lymphoma, vascular leukoencephalopathy and multiple cerebral infarcts). These findings indicate that the presence of pTDP-43 aggregates in skeletal muscles occurs in various conditions that may or may not include NMDs.

In the present study, we have demonstrated that the mean density of pTDP-43 aggregates in ALS was significantly higher than that in NMDs and non-NMDs. Since TDP-43 is an RNA binding protein ubiquitously expressed in all tissues, it is possible that cytoplasmic TDP-43 aggregates may occur in the degenerating or regenerating muscle fibers. Vogler et al. suggested that increased assembly or decreased clearance of functionally normal myo-granules, one of stress granules in myofibers, could be the source of cytoplasmic TDP-43 aggregates in regenerating muscle fibers [24]. However, it is enigmatic that there is a difference in the incidence of pTDP-43 aggregates between ALS and NMDs or non-NMDs. In the present study, pTDP-43 aggregates were also immunopositive for p62, one of markers for autophagy process, indicating that pTDP-43 aggregates are degraded by the autophagic process. The ability of protein degradation by autophagy is variable depending on the conditions of tissue and/or cells [5]. Differential autophagy power could be a possible reason for higher density of pTDP-43 aggregates in ALS as a multisystem proteinopathy.

To our knowledge, this is the first time that pTDP-43 aggregates have been reported in the myocardium, being present in patients with ALS (12 of 30 cases), NMDs (4 of 13 cases) and non-NMDs (1 of 7 cases). Since atrophy of the myocardium is not a neurogenic process, the presence of pTDP-43 aggregates in cardiac muscle may imply the involvement of a myogenic mechanism. With regards to the abnormal protein deposition in cardiac tissues of neurodegenerative diseases, phosphorylated α-synuclein aggregates are observed in the cardiac sympathetic nerves, but not in the cardiac myofibers, in Lewy body diseases [18]. In the present study, pTDP-43 aggregates were not noted in the peripheral nerve elements in any of the patients with ALS, NMDs or non-NMDs. Moreover, in contiguous sections stained with HE and anti-pTDP-43, skeletal and cardiac muscles with dense filamentous inclusions were shown to exhibit single-fiber atrophy with vacuolar degeneration, suggesting that pTDP-43 aggregates are associated with myogenic degeneration in skeletal and cardiac muscles. With regards to cardiac muscle injury, it is known that the plasma level of cardiac troponin, a biomarker of myocardial injury, is elevated in ALS. pTDP-43 aggregates with vacuolar degeneration in cardiac muscles might be related to the plasma level of cardiac troponin in ALS patients.

The present study revealed two types of pTDP-43 aggregates (dense filamentous and short linear inclusions) in skeletal and cardiac muscles. Both types were also immunopositive for nTDP-43 and p62. These immunohistochemical features are similar to those of pTDP-43 aggregates (skein-like and round inclusions) found in the central nervous system of ALS patients [11, 13, 14]. Moreover, treatment with PK reduced the number of short linear inclusions, whereas dense filamentous inclusions were PK-resistant, suggesting that the latter are aggregated more strongly than short linear inclusions. Previously, we demonstrated that in ALS linear wisps (“fine skeins”) aggregate as thicker and longer threads, leading to the formation of skein-like inclusions in the spinal anterior horn cells [14]. Based on the above findings, we assumed that the maturation process of dense filamentous inclusions is divided into several stages (Fig. 4d, g, j). At first, short-linear aggregates of pTDP-43-immunoreactive material appear diffusely in the cytoplasm (1 in Fig. 4d). Then, short-linear aggregates accumulate in the juxtanuclear cytoplasm with small vacuoles (2 in Fig. 4g). The next stage is observed as small dense filamentous aggregates in small vacuoles (3 in Fig. 4g). Finally, larger dense filamentous aggregates are found in a dilated vacuole (4 in Fig. 4j). pTDP-43-positive profiles (short-linear and dense filamentous aggregates) are also immunoreactive for nTDP-43 and p62 (Fig. 4d-l). p62 is one of the markers for autophagy process, which labels not only pathological cytoplasmic aggregates but structures related to the autophagy process. Autophagy participates in degradation process of abnormal proteins. Recently, we demonstrated that autophagy is a common degradation pathway for TDP-43 inclusions in ALS [12]. These findings suggest that short linear inclusions may be precursor inclusions.

The density of pTDP-43 aggregates in skeletal and cardiac muscles was very low in our samples, for example 1–2 aggregates per 1 cm^2^ and the size of pTDP-43 aggregates in our samples was very small. Judging from the light microscopic findings reported by Cykowski et al. [6], pTDP-43 aggregates are much abundant and their size seems to be larger in Cykowski’s report. Although the methods of technique and tissue handling we used are different from Cykowski’s methods, we feel that these morphological differences may be attributable to environmental and/or genetic factors unique to the American and Japanese populations. Further investigations will be necessary to clarify the factors responsible for variations in the extent of pTDP-43 pathology in muscles.

## Conclusions

The present findings suggest that pTDP-43 aggregates in skeletal and cardiac muscle are a marker of myogenic degeneration in amyotrophic lateral sclerosis and various conditions associated or unassociated with NMDs.

## Data Availability

All the data generated or analyzed during this study are included in this published article.
